# Magnetic Resonance Imaging-Guided Adaptive Radiotherapy for Colorectal Liver Metastases

**DOI:** 10.3390/cancers13071636

**Published:** 2021-04-01

**Authors:** Paul B. Romesser, Neelam Tyagi, Christopher H. Crane

**Affiliations:** 1 Department of Radiation Oncology, Memorial Sloan Kettering Cancer Center, New York, NY 10065, USA; romessep@mskcc.org; 2 Early Drug Development Service, Department of Medicine, Memorial Sloan Kettering Cancer Center, New York, NY 10065, USA; 3 Department of Medical Physics, Memorial Sloan Kettering Cancer Center, New York, NY 10065, USA; tyagin@mskcc.org

**Keywords:** colorectal liver metastasis, stereotactic ablative radiation therapy, proton therapy, MRI guided radiotherapy

## Abstract

**Simple Summary:**

Stereotactic ablative body radiotherapy (SABR) is an effective treatment for CRC liver metastases. Advanced radiation techniques are required to ensure safe and efficacious treatment. MR-guided radiotherapy is a new and evolving technology that can overcome many of the challenges of liver SABR. MR imaging before, during and after treatment delivery facilitates direct visualization of tumor target and adjacent normal healthy organs, real time MR imaging facilitates non-invasive tumor tracking and gating, and daily adaptive re-planning permits treatment plans to be adjusted for the anatomy of the day. MR-guided ablative radiation therapy is a promising radiation technology advance that can further facilitate safe tumor dose escalation of colorectal liver metastases.

**Abstract:**

Technological advances have enabled well tolerated and effective radiation treatment for small liver metastases. Stereotactic ablative radiation therapy (SABR) refers to ablative dose delivery (>100 Gy BED) in five fractions or fewer. For larger tumors, the safe delivery of SABR can be challenging due to a more limited volume of healthy normal liver parenchyma and the proximity of the tumor to radiosensitive organs such as the stomach, duodenum, and large intestine. In addition to stereotactic treatment delivery, controlling respiratory motion, the use of image guidance, adaptive planning and increasing the number of radiation fractions are sometimes necessary for the safe delivery of SABR in these situations. Magnetic Resonance (MR) image-guided adaptive radiation therapy (MRgART) is a new and rapidly evolving treatment paradigm. MR imaging before, during and after treatment delivery facilitates direct visualization of both the tumor target and the adjacent normal healthy organs as well as potential intrafraction motion. Real time MR imaging facilitates non-invasive tumor tracking and treatment gating. While daily adaptive re-planning permits treatment plans to be adjusted based on the anatomy of the day. MRgART therapy is a promising radiation technology advance that can overcome many of the challenges of liver SABR and may facilitate the safe tumor dose escalation of colorectal liver metastases.

## 1. Introduction

Colorectal cancer commonly metastasizes to the liver and can often be localized there. In the past, liver metastases were thought to be incurable and the only treatment option considered was systemic cytotoxic chemotherapy [1]. The treatment algorithm for colorectal cancer patients with disease confined to the liver has changed dramatically over the prior 20 years, with the advent of highly effective therapies to treat liver metastases [2]. The recognition of the oligometastatic state, defined as limited but not widespread metastatic disease, has helped spur further interest in developing effective local therapies to treat metastatic tumors [3].

Surgical metastasectomy series provided the initial data to support the benefit of metastatic directed treatments. Multiple groups have reported prolonged progression-free survival (PFS) and overall survival (OS) following metastasectomy for liver and/or lung metastases in patients with oligometastatic colorectal cancer [4,5,6,7,8,9,10,11,12,13]. Re-analysis of the Intergroup 0114 study noted an improvement in OS in rectal cancer patients with isolated metastatic disease treated with surgical resection as compared to no surgical resection (5 year OS 27% vs. 6%, *p* < 0.001) [14]. Review of patients with newly diagnosed metastatic CRC (mCRC) treated at MD Anderson Cancer Center and Mayo Clinic demonstrated improvements in OS, for patients treated between 1998 and 2004, which was attributed primarily to an increase in hepatic resection [15]. Additional large retrospective series have demonstrated 5-year OS rates of nearly 40% in colorectal cancer patients with isolated liver metastasis following resection [4,5,9]. For patients with limited extrahepatic disease, complete surgical resection has also been associated with prolonged survival [13]. Shah et al. reported median disease-free survival and OS durations of 19.8 and 87 months in 39 patients who underwent both lung and liver resections for mCRC [11]. The 5-year OS was significantly longer for patients who underwent both liver and lung resections than those who did not undergo lung resection for pulmonary metastases (74% vs. 42%, *p* = 0.05) [11]. These data, when taken together, demonstrate that for selected patients with oligometastatic CRC surgical metastasectomy can dramatically alter disease progression.

While surgical metastasectomy remains the standard of care, only ~20–30% of patients are surgical candidates [16]. Thus, there is interest in non-surgical treatment schemas, such as interventional radiology (IR) ablation with radiofrequency or microwave techniques or even high-intensity focal ultrasound and cryotherapy [17]. In fact EORTC 40004 demonstrated improved PFS and OS for radiofrequency ablation (RFA), as compared to systemic chemotherapy, in a randomized phase II trial for colorectal cancer patients with surgically unresectable disease limited to the liver [18,19]. Not all patients are good candidates for IR ablation as tumors in close proximity to large blood vessels and large tumors (i.e., >3 cm) have been reported to have worse local control [20,21]. IR ablation is safe, effective, and convenient for appropriate selected patients [20].

Stereotactic ablative body radiotherapy (SABR) is a noninvasive alternative treatment option. The safety of SABR for the treatment of 2 to 4 extracranial metastases has been demonstrated in the cooperative group setting with NRG-BR001, a phase I trial. Preliminary data indicate that SABR to multiple metastatic sites in the liver, lung, abdomen/pelvis, bone, and spine is safe [22]. No dose-limiting toxicity was identified across the evaluable anatomic locations [23,24]. The efficacy of SABR for treating oligometastatic disease was evaluated in SABR-COMET, which was a randomized, phase II tumor agnostic trial in 99 patients with up to 5 metastatic lesions [25]. Of the patients enrolled in the trial, 27% in the control group and 14% in the SABR group had a CRC primary. The trial met its primary endpoint of improved OS, with a median OS of 41 months in the SABR arm versus 28 months in the control arm (HR 0.57, 95% CI 0.30–1.10, *p* = 0.090). While 4.5% of patients in the SABR group had grade 5 treatment-related adverse events, the 5-year OS rate was 17.7% in the control arm as compared to 42.3% in the SABR arm [26]. Given these data, the use of SABR has gained popularity as ~70% of radiation oncologists reported using SABR for the treatment of oligometastatic disease [27].

## 2. The Evolution of External Beam Radiation Therapy for Liver Tumors

Historically, external beam radiation therapy for the treatment of liver metastases was reserved for patients requiring palliation of painful liver disease. While, whole liver radiation (WLR) can palliate symptoms, WLR is ineffective at eradicating metastatic disease given the risk for radiation induced liver disease (RILD) [21,28,29,30]. The severity of RILD depends on a variety of factors including radiation dose and fractionation, volume of liver irradiated, and underlying liver dysfunction [31].

To reduce the risk for RILD, the Quantitative Analyses of Normal Tissue Effects in the Clinic (QUANTEC) published recommendations that restricted the volume and dose for whole liver radiation to 30 Gy in 2 Gy fractions or 21 Gy in 3 Gy fractions [32]. As the risk of RILD is correlated with the dose and volume of liver irradiated these constraints limit the risk of RILD to less than 5% [32]. As these constraints precluded doses required to eradicate liver disease many prematurely concluded that radiation therapy had a limited role in the treatment of intrahepatic malignancies and metastases [33].

Advances in radiotherapy technology provided the opportunity to develop techniques to deliver very high radiation doses to partial liver volumes, while simultaneously sparing healthy liver parenchyma [32]. Given these data, QUANTEC updated the practice guidelines for SABR to the liver. Current dose constraints recommend limiting the mean radiation dose to the liver to <15 Gy in 3 fractions and limiting 700 cm^3^ of functional healthy liver to <15 Gy in 3–5 fractions [32]. These new constraints were developed to limit the risk of RILD. In patients with chronic liver disease or advanced cirrhosis the risk for RILD is higher and more stringent constraints should be employed [34,35,36,37,38,39,40]. In addition, it is important to optimize liver function and address any reversible causes of liver decompensation prior to liver SABR.

The Karolinksa Institute was the first to report the efficacy and safety of high dose focal liver irradiation using a stereotactic technique in the 1990’s [41]. Additional centers have prospectively reported on the efficacy and safety of liver SABR, Table 1 [42,43,44,45,46,47,48,49,50,51,52,53,54,55]. Our prior review provides more depth on the background and history of the liver SABR [3]. Importantly, there seems to be a correlation between tumor control and radiation dose [42,56,57]. As the treatment of liver metastases is a balance between adequately ablating the tumor while protecting the normal healthy liver, the future will likely entail tailoring radiation dose based on the predicted tumor radiation sensitivity to achieve 90% local control while limiting the risk of RILD to less than 5% or more appropriately as low as possible.

## 3. Liver SABR Treatment Planning and Delivery Considerations

Liver SABR has the potential for serious side effects if the delivery does not accurately reflect the treatment plan. All patients undergo standard simulation scan in the treatment position with computed tomography (CT). These images are used for treatment planning. As accurate planning requires tumor visualization, the simulation CT scans typically includes intravenous contrast administration as deemed necessary to help facilitate tumor and normal organ delineation. In addition, alternative imaging modalities (positron emission tomography (PET) or magnetic resonance imaging (MRI)) can be utilized to ensure accurate delineation of the tumor target and adjacent organs at risk (OAR) [21,58,59].

To localize the tumor and to protect the radiosensitive adjacent normal tissues such as the stomach, small intestine, and large intestine, respiratory motion must be controlled or accounted for during simulation and treatment delivery. The degree of movement, especially near the liver dome, can be significant [60]. Multiple strategies can be employed to either control or account for tumor motion. One relatively straightforward technique to account for tumor motion is to include a four-dimensional CT (4DCT) with the radiation simulation scan. The 4DCT will allow liver and, more importantly tumor, motion to be accounted for throughout the respiratory cycle. Including the path of tumor movement in the treatment target volume ensures adequate coverage, albeit at the cost of more liver parenchyma being treated given the larger margin around the tumor. Thus, the use of a 4DCT is most appropriate for small tumors that are far away from radiosensitive luminal organs. More advanced motion management techniques deliver treatment during specific phases of respiration. These techniques are known as gating [61,62]. Gating can also use deep inspiratory breath hold (DIBH), where patients are treated while holding near maximal inspiration. For DIBH to be successful patients must be relatively fit and able to hold their breath for 20–40 s intervals. Similar in concept to DIBH is end-expiratory gating. Here, patients are breathing normally, but the delivery of radiation is restricted to the expiration phase of the natural breathing cycle. An alternative strategy is abdominal compression. Abdominal compression treats throughout the respiratory cycle, but the amplitude of the respiratory cycle is restricted, which limits tumor motion to generally less than 5 mm. With abdominal compression a cuff is placed around the abdomen of the patient and insufflated to restrict respiratory motion. As pressures can reach 40–50 mmHg, some patients find abdominal compression to be uncomfortable and anxiety provoking. While the goal of compression is to limit diaphragmatic movement, it also compresses the radiosensitive abdominal organs into the liver. Thus, abdominal compression may not be optimal in all cases and is of particular concern for metastases in the left lobe, which is in close proximity to the stomach, but generally not a concern for metastases in the right lobe [63]. All of these motion management techniques have advantages and disadvantages that need to be considered when determining the most appropriate technique for an individual patient. In addition, most centers and radiation oncology teams only have experience with one or two of these motion management techniques and limited experience with the others, which makes cross comparing them difficult.

For accurate treatment delivery the patient and tumor must be accurately aligned daily for treatment. As the position of the liver in relation to bony anatomy can change by up to 1 cm between fractions, liver SABR presents a particular challenge [64,65]. Since metastatic tumors are difficult to visualize fiducial markers or the shape of the liver can be used to help ensure proper alignment with daily non-contrast cone beam CT (CBCT) scans taken with the patient in the treatment position on the treatment machine [66]. While setting up to liver shape is a significant improvement as compared to aligning relative to bony anatomy, there is still a degree of inter-fractional motion uncertainty especially at the dome of the liver. For tumors near the dome of the liver, it is preferable to add an additional margin cranially and caudally due to the variability of the position of the diaphragm with DIBH if the alignment will be to the liver shape. One way to improve daily tumor localization and decrease inter-fraction motion is to place fiducial markers in and around the tumor. Alternatively, surgical clips can be utilized if already in place and in close proximity to the tumor [67,68,69]. Fiducial markers and/or appropriate positioned surgical clips can help ensure target accuracy (~2–3 mm) with real time intrafraction motion assessment [58]. A well utilized strategy for motion management will allow smaller margins for treatment and help ensure the safety and efficacy of SABR for tumors in close proximity of radiosensitive OARs, as well as large tumors where the dose to the healthy liver parenchyma is limiting.

## 4. Strategies to Overcome Limitations to SABR

SABR to liver metastases can be challenging in patients with large tumors and/or tumors abutting or in close proximity to radiosensitive gastrointestinal organs [70]. When limited by adjacent OARs, radiation oncologists commonly opt to de-intensify treatment to a sub-ablative palliative dose. This strategy while safe, precludes tumor ablation in patients who have a high likelihood of benefit. Proceeding with sub-ablative treatment, fails to meet the goal of oligometastatic directed therapy. An alternative approach is to consider adopting moderate hypofractionation (e.g., expanding to 10, 15, or even 25 fractions) to maintain an ablative treatment [3]. Fractionation results in greater normal tissue tolerance as sublethal DNA damage is repaired between fractions. Moderate hypofractionation maintains an ablative dose, while prioritizing safety (i.e., limits risk of GI bleeding, RILD, and bile duct strictures) [69]. If OAR constraints cannot be met for a tradition hypofractionated SABR plan one must decide to either proceed with an unsafe treatment to maximize tumor control, proceed with a safe treatment that compromises tumor control, or alternatively to adopt moderate hypofractionatation to maintain tumor control while ensuring safety. As we have described previously, we strongly favor adopting moderate hypofractionation to ensure safety while maximizing tumor control [3].

In patients with large tumors or underlying liver dysfunction, proton beam radiotherapy should be considered. Protons come to rest and deposit their energy at a prespecified range in the patient, the Bragg peak. As there is minimal exit dose, protons have a dosimetric advantage over photon radiotherapy. Thus, proton SABR for liver metastases can spare more normal liver than photon SABR [71]. In some patients with bi-lobar or large tumors where the liver dose constraints will be impossible to meet with photon based SABR, protons may permit ablative radiation therapy [72]. In fact, patients with primary liver tumors treated with proton, as compared to photon, radiotherapy resulted in less liver decompensation and better survival [73]. This survival benefit was attributed to a significantly lower mean liver dose, which decreased the risk of non-classic RILD in proton patients [73]. Additional prospective clinical trials have demonstrated the efficacy and safety of proton liver SABR [51,53]. Just as with photon based therapy, considerations need to be taken for motion management and image guidance with proton radiotherapy.

## 5. MR Guided Adaptive Radiation Therapy

MRgART is a promising radiation technology advance that can overcome many of the challenges of traditional liver SABR and may facilitate safe tumor dose escalation of colorectal liver metastases. The US Food and Drug Administration (FDA) has recently approved the Elekta Unity^TM^ (Elekta, Stockholm, Sweden) and the View Ray MRIdian^TM^ (ViewRay, Oakwood Village, OH, USA) MRI-guided radiation treatment systems for clinical use. Unlike traditional image guidance which use on-board CBCT, the coupling of a MRI and treatment LINAC permits image acquisition before, during, and after radiation delivery, with the patient in the treatment position [74].

**Figure 1:**
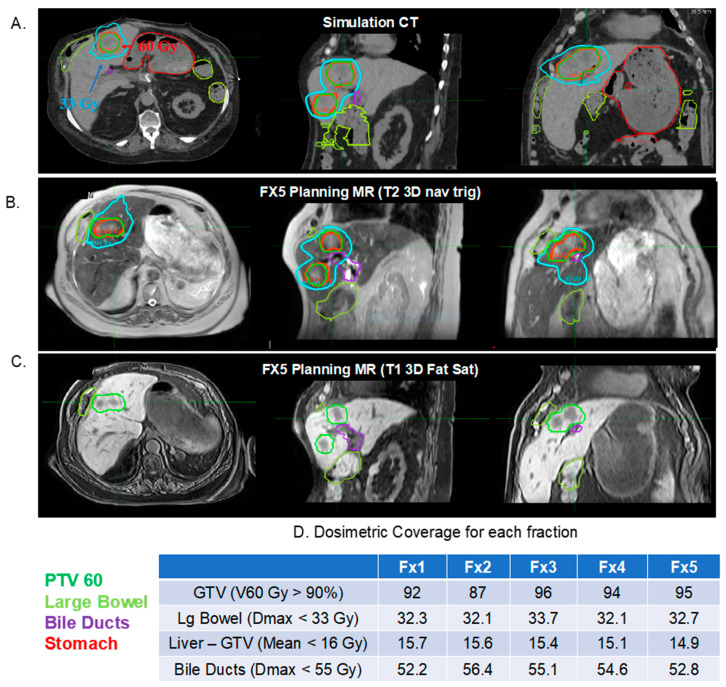
MR-guided adaptive radiation therapy. Seventy-two-year-old female with three liver metastases (2 cm lesion in segment 8 and segments 3/4B measuring 1.6 cm) treated using MRg-ART on Elekta Unity MR-linac to a prescription of 60 Gy (12 Gy in 5 fractions). (**A**) The tumor metastasis was not visible on non-contrast planning CT as the patient was not a candidate for iodinated IV contrast due to poor renal function. Patient was simulated supine in a custom immobilization device and with an abdominal compression belt to minimize respiratory liver motion to <5 mm. (**B**) Online adaptive plan for a fraction on a T2 3D navigator triggered MRI. (**C**) T1w 3D fat saturated MRI was used in combination with T2 3D navigator triggered sequence to delineate GTV and OARs during online planning. Daily adaptive planning was performed to account for coverage of multiple lesions (due to varying deformations in the liver) as well as to protect the organs at risk. Bile ducts were used as the monitoring structure on the three orthogonal plane balanced fast field echo cine MRI during radiation delivery. (**D**) Daily dosimetric coverage for GTV and various relevant OARs. Each fraction dose is displayed in terms total prescription dose.

The addition of on-board MR imaging offers a number of advantages as MR imaging can be done to ensure patient alignment before treatment, as with typical CBCT-guided approaches, albeit without the excessive radiation, and also continuously during treatment delivery. First, MR imaging offers superior soft tissue delineation so that liver metastases can be accurately visualized and thus alleviate the need for multi-phase contrast CT simulation scans (Figure 1A). In fact, many groups are working on MR-based radiation planning and thus avoiding CT based simulations altogether [75,76]. Second, dynamic MR imaging during before, during, and after treatment allows temporospatial tumor monitoring throughout treatment to ensure accurate targeting and treatment delivery [77]. The combination of superior contrast resolution combined with a fiducial less treatment is a significant advantage over CBCT scans obtained on traditional LINACs for image guided SBRT [3]. Given the superior soft tissue delineation with MRI, liver tumors can be readily identified and patients can thus be treated without fiducials and spared an invasive and now unnecessary procedure. Similar to non-MR guided treatment, motion management must be accounted for. Many of the same techniques discussed above (i.e., DIBH, gating, and abdominal compression) can be used with MR-radiotherapy systems but require special MR compatible equipment. One potential significant advance is MR based tumor tracking and gating. As the tumor is monitored in real time with dynamic MR imaging, treatment can be delivered only when the tumor is within a pre-specified treatment gate [77]. This improved accuracy decreases inter- and intra-fraction motion and permits treatment with tighter margins, thus sparing more healthy viable tissue. Third, MR-guided treatment programs permit adaptive re-planning, daily in real time, to account for inter-fractional tumor and normal organ motion (Figure 1B–D) [78]. Changes to the radiation plan can be made on a daily basis to ensure optimal tumor target and normal tissue sparing. Much of the aforementioned limits of SABR are easily overcome with MR-guided based treatment as MR imaging permits accurate tumor localization thus minimizing inter-fraction motion and alleviating the need for fiducial marker placement, dynamic imaging during treatment delivery provides a non-invasive system to adapt for tumor movement with respiration, and rapid adaptive replanning in real time provides an opportunity to adjust the radiation delivery plan daily depending on changes in internal anatomy.

Initial experiences in using MRgART for primary and metastatic liver tumors have been published [79,80,81,82]. Rosenberg et al. reported a multi-institutional retrospective series on 26 patients with liver tumors (45% colorectal liver metastasis) treated with MRgART to a median dose of 50 Gy in 10 Gy fractions [79]. Freedom from local progression was 80% at 21 months (median follow up). Toxicity was low with 7.7% grade 3 gastrointestinal toxicity and no grade 4+ toxicity appreciated. Interestingly, Rosenberg et al. postulate that traditional dose and volume constraints for organs at risk might be overly conservative for MR-based radiotherapy given the significantly improved tumor and normal tissue delineation and temporospatial real time monitoring [79]. Clinical trials are underway to better define normal tissue dose constraints in the MR-guide radiotherapy era [79].

At our institution, MRgART for Liver SABR is performed using Elekta’s Adapt-to-Shape workflow to a prescription of 54–60 Gy in 3–5 fractions [78]. All patients are simulated and treated with abdominal compression to reduce both tumor and OAR motion to be less than 5 mm. Automatic breath-hold and gating or other motion management options are not currently available on the Unity MR-linac system. The GTV volumes on simulation CT are drawn with the help of MR simulation images that includes a 2D T2w turbo spin echo MR (TR/TE = 1250/80 ms, slice thickness = 4 mm, voxel size = 1.3 × 1.6 mm^2^) and a 3D radial T1w acquisition called 3DVANE radial MR sequence (TR/TE = 4.1/1.68 ms, slice thickness = 4 mm, voxel size = 1.4 × 1.4 mm^2^) acquired after Eovist contrast injection. During each treatment fraction, a 3D T1w fat saturated MRI (TR/TE = 4.6/2.3 ms, voxel size = 2 × 2 × 2 mm^3^, FOV = 450 × 450 × 250 mm^3^) and/or T2 3D navigator triggered (TR/TE = 1900/87 ms, voxel size = 2 × 2 × 2.4 mm^3^, FOV = 400 × 448 × 250 mm^3^) sequence is registered to the reference CT/MR by first performing a rigid spine match and then adjusting the fusion to match the GTV. The remaining OARs are propagated using Monaco deformable image registration algorithm and manually edited by the physician and planners. The simulation CT and on-treatment planning MRIs, both represent a motion averaged image of the residual motion with the compression belt. A new adaptive plan is generated in Monaco using fluence optimization. Right before beam-on, another 3D T1w fat saturated MRI is acquired, and the structures and plan-of-the-day are overlaid to assess GTV coverage and evaluate for any potential intrafraction motion that may have occurred during contouring and planning. The contours are adjusted, and a new plan is generated if significant motion occurs. Otherwise, the treatment is delivered using a three orthogonal plane 2D balanced fast-field echo cine MR monitoring at 5 frames/s (TR/TE = 2.6/1.32 ms, slice thickness = 5 mm, FOV = 400(FH) × 424(RL) mm^2^, voxel size = 3 × 3 mm^2^) at the centroid of the motion monitoring structure. Our team typically make use of an OAR near the high dose PTV (e.g., stomach or bile ducts) as the monitoring structure. Overall average adapt-to-shape time divided into contouring, planning, physics QA and beam-on time is 21.1 ± 7.2 min, 14.5 ± 5.2 min, 4.4 ± 1.0 min and 14.7 ± 2.2 min, respectively.

As MRgART is a new technology, there remain limitations that need to be overcome. Contouring of gross tumor volumes (GTV) and OARs is still one of the most time and resource consuming part of online MRgART planning and require the use of MR-based auto-segmentation algorithms in routine clinical workflow [83]. Due to the lack of robust clinical tools, coverage to OARs are still assessed based on a conservative assumption that the maximum dose lies in the same position from one fraction to another. Clinical tools that can accurately estimate both the spatial and temporal variation of the maximum doses to the OARs will allow further safe delivery of ablative doses to the target by eliminating the need to over-constrain the OARs by adding larger safety margins. Deformable image registration based dose accumulation studies for MRgART are limited and is an area of active development [84]. Current clinical workflows for MRgART allow use of single and limited MR sequences for contouring GTV and OARs during online planning. Alternative workflows on Elekta Unity are emerging that can make use of multiple MR contrast simultaneously [85]. Figure 1 shows the use of T2w 3D navigator triggered MR that was used simultaneously with T1 3D fat saturation MRI for GTV and OAR delineation. Finally, liver lesions by themselves may not be visible on the vendor provided single or multiple orthogonal plane 2D cine monitoring MR scan and may require the use of MRI contrast agent such as Eovist or surrogate structures such as liver or bile ducts for patient position monitoring or gating. Development of real-time volumetric MRI for online planning and tumor tracking will further improve the accuracy of adaptive treatments for GI tumors [86].

**Figure 2:**
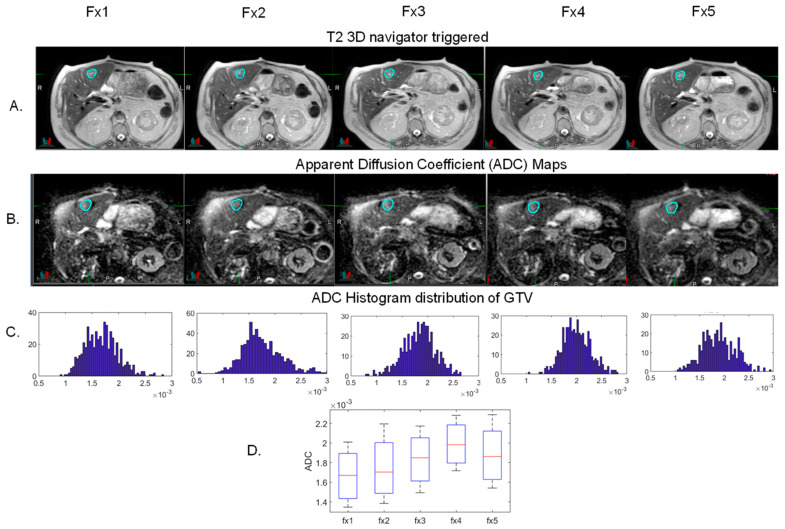
Treatment response of an example liver lesion using T2w MRI and diffusion weighted MRI derived apparent diffusion coefficient maps. (**A**) T2w 3D navigator triggered MRI for during treatment fractions with GTV lesion displayed. (**B**) Apparent diffusion coefficient maps derived using four b-values (0, 30, 150 and 550 mm^2^/s). (**C**) Histogram distribution of ADC values within the GTV. (**D**) Trend analysis of ADC values using boxplot.

Beyond improving clinical outcomes, functional MR imaging before, during, and after treatment may lead to the identification of predictive radiomic markers of response [87]. The MR-guided systems can acquire functional images and assess changes in the tumor and tumor microenvironment during and after treatment and provide an ideal platform to generate reproducible quantitative biomarkers and facilitate collection and validation of large datasets for use in outcome research. Initial pilot studies performed on these systems have shown that such measurements are feasible [88,89,90,91]. This will hopefully pave the way for more personalized therapies as our understanding of tumor histology, size, genetics, and radiomics continues to improve. Figure 2 shows the treatment response assessment of one of the liver lesions shown in Figure 1 using apparent diffusion coefficient (ADC) derived from diffusion weighted (DW-) MRI. These images were acquired on the Elekta Unity platform using four b-values (0, 30, 150 and 550 mm^2^/s). The histogram distribution as a function of treatment fraction shows increasing number of pixels shifting towards high ADC values. The increasing ADC trend over the course of treatment suggest good tumor response. Follow up imaging studies will corroborate that. Studies in various tumor subsites have suggested that increase in ADC is a surrogate for good tumor response [92,93,94]. Multiple groups have just started to acquire such data on their MR-guided systems without adding any additional time for acquisition. Eventual goal of this data will be to correlate during treatment response to long term tumor control.

## 6. Conclusions

SABR is an effective treatment for CRC liver metastases with locoregional control rates in prospective clinical trials in excess of 75%. Advanced radiation techniques are required to ensure safe and efficacious treatment. In patients with large liver metastases or in metastases abutting or in close proximity to radiosensitive gastrointestinal organs SABR can be challenging. Motion management and consideration for moderate hypofractionation treatment schedules are important to ensure efficacy and safety of liver SABR. For patients with exceptionally large tumors, limited liver reserve, or underlying chronic liver disease proton radiotherapy should be considered. MR-guided radiotherapy is a new and evolving technology that can overcome many of the challenges of liver SABR. MR imaging before, during and after treatment delivery facilitates direct visualization of tumor target and adjacent normal healthy organs, real time MR imaging facilitates non-invasive tumor tracking and gating, and daily adaptive re-planning permits treatment plans to be adjusted for the anatomy of the day. MR-guided ablative radiation therapy is a promising radiation technology advance that can further facilitate safe tumor dose escalation of colorectal liver metastases.

## Figures and Tables

**Table 1:** Liver SABR prospective trials.

Study	Study Type	Patient #	Lesion #	Primary Histology	Dose (Gy)/ Fraction Number	BED (a/b = 10)	Toxicity	Local Tumor Control	Overall Survival
Herfarth et al. [42]	Phase I/II	37	60	Mixed	14-26/1	33–94		71% @ 1 year	72% @ 1 year
Mendez–Romero et al. [43]	Phase I/II	17	39	Mixed	37.5/3	84	12% G3 Liver	100% @ 1 year 86% @ 2 years	85% @ 1 year 62% @ 2 years
Rusthoven et al. [44]	Phase I/II	47	63	Mixed	36–60/3	79–180	1.5% G3 (dermatitis)	95% @ 1 year 92% @ 2 years	30% @ 2 years
Lee et al. [45]	Dose escalation, phase I	68	142	Mixed	28–60/6	41–120	10% G3+	71% @ 1 year	47% @ 1.5 years
Rule et al. [46]	Dose escalation, phase I	27	37	Mixed	30/3–50–60/5	60–132	4% G3 Liver	100%, 89%, 56% * @ 2 years	50%, 67%, 56% * @ 2 years
Comito et al. [47]	Observational	42	52	Colorectal	75/3	263	60% G2, 0%G3	95% @ 1 year 85% @ 2 years	85% @ 1 year 65% @ 2 years
Scorsetti et al. [48]	Phase II	42	52	Colorectal	75/3	263	25% G2 Liver, 0% G3	95% @ 1 year 91% @ 2 years 85% @ 3 years	65% @ 2 years
Goodman et al. [49]	Dose escalation, phase I	26	40	Mixed	18–30/1	50–120	8% GI bleeding	77% @ 1 year	50% @ 2 years
Meyer et al. [50]	Dose escalation, phase I	14	17	Mixed	35–40/1	158–200	6% G2	100% @ 2.5 years	78% @ 2 years
Hong et al. [51]	Phase II	89	143	Mixed	30–50/5	48–100	No G3+	72% @ 1 year 61% @ 3 years	66% @ 1 year 21% @ 3 years
Scorsetti et al. [52]	Phase II	61	76	Mixed	75/3	263	2% G3 chest wall pain	94% @ 1 year 78% @ 3 years 78% @ 5 years	85% @ 1 year 31% @ 3 years 18% @ 5 years
Kang et al. [53]	Phase I	9	14	Mixed	36–60/3	79–180	No G3+	NR	NR
Dawson et al. [54]	Dose escalation, phase I, multicenter	26	37	Mixed	35–50/10	47–75	7.7% G3 GI	NR	NR

# = number, cm = centimeter, BED = biologically effective dose, Gy = Gray, NR = not reported, GI = gastrointestinal, G3 = grade 3, G2 = grade 2, @ = at, * = 60 Gy/12 Gy per fraction, 50 Gy/10 Gy per fraction, and 30 Gy/10 Gy per fraction dose cohorts.

## Data Availability

No new data were created or analyzed in this study. Data sharing is not applicable to this article.
